# Feature representation for explainable CRISPR off-target prediction and base editing efficiency

**DOI:** 10.3389/fbinf.2026.1800237

**Published:** 2026-04-20

**Authors:** Faiza Hasin, Michele Minervini, Corrado Mencar, Giuseppe Ventrella, Arianna Consiglio, Alessandro Orro, Tommaso Selmi

**Affiliations:** 1 Department of Computer Science, University of Bari Aldo Moro, Bari, Italy; 2 Institute for Biomedical Technologies (Bari Unit), National Research Council (CNR), Bari, Italy; 3 Institute for Biomedical Technologies (Milan Unit), National Research Council (CNR), Milan, Italy

**Keywords:** base editing, CRISPR/Cas9, explainability, feature representation, off-target prediction, shap, XGBoost

## Abstract

**Introduction:**

The interaction between guide RNAs (gRNAs) and target DNA sequences is a critical factor in the effectiveness of CRISPR/Cas9 (Clustered Regularly Interspaced Short Palindromic Repeats/CRISPR-associated protein 9) gene editing. Predicting these interactions accurately necessitates models that offer biological knowledge in addition to high accuracy. This study analyzes the impact of feature representation on accuracy and interpretability in off-target prediction.

**Methods:**

We address two CRISPR applications: gene knockout (KO) and base editing (BE) using distinct benchmark datasets. For the KO problem, we utilized CHANGE-seq and GUIDE-seq to evaluate paired sequence representations, while the Hanna screening dataset has been used for BE. We approached the prediction problem both as a classification and regression task using XGBoost models.

**Results:**

In the case of KO, there is not a single universally optimal encoding. For both classification and regression, One-Hot and its variants (OH, OH5C) achieve the best results on GUIDE-seq (AUPR = 0.661, Pearson = 0.756), while the Bulges representation performs best on CHANGE-seq (AUPR = 0.612, Pearson = 0.602). In the case of BE, One-hot encoding consistently outperforms K-mer representation for predictive accuracy both as regression and classification (AUPR = 0.723, Pearson = 0.746).

**Discussion:**

Our analysis demonstrates comparable predictive performance across both gene knockout and base editing tasks, confirming the robustness of the framework in distinct editing domains. Interpretability analysis using SHapley Additive exPlanations (SHAP) reveals that despite different mechanisms, the Protospacer Adjacent Motif (PAM)-proximal region remains a critical feature for prediction for both editing mechanisms.

## 1 Introduction

CRISPR/Cas9 (Clustered Regularly Interspaced Short Palindromic Repeats/CRISPR-associated protein 9) is an innovative genome-editing technique that allows researchers to fix genetic mutations, disable detrimental genes, or add advantageous features. It achieves this by targeting and editing specific DNA regions using a customizable guide RNA (gRNA), which directs the Cas9 endonuclease to act as molecular scissors (Anders et al., 2014). One main obstacle of CRISPR/Cas9 technology concerns unwanted genetic alterations that may result from inadvertent alterations in off-target genomic locations due to several concurring causes, which could compromise research results and raise safety issues in therapeutic outcomes (Zhang et al., 2019).

Advanced computational methods, notably Machine Learning (ML), are now crucial for predicting off-target effects (Yaish et al., 2022). Specifically, Gradient Boosting has demonstrated significant effectiveness in enhancing the precision of predictive models due to its capability to capture complex, nonlinear data relationships (Chen and Guestrin, 2016). Nonetheless, selecting an appropriate data representation is vital, as it influences the model’s performance by differentially enhancing the relationships between features, thereby affecting the ML models’ predictive accuracy. Moreover, the selection of representation plays a crucial role in determining the model’s interpretability, particularly regarding aligning the model’s decisions with biologically comprehensible data. Among these techniques, XGBoost has been widely adopted due to its strong predictive performance, computational efficiency, and ability to model nonlinear interactions between features. Previous studies have shown that gradient boosting models perform particularly well on structured biological datasets and often outperform traditional machine learning models while maintaining interpretability. In CRISPR-related prediction tasks, several studies highlight the effectiveness of XGBoost and other tree-based ensemble methods for modeling complex sequence–activity relationships Saraswat and Ranjan (2025).

However, beyond algorithm choice, one of the most decisive factors in model quality is feature representation—that is, how nucleotide sequences are encoded before being passed to a learning algorithm (Shrikumar et al., 2017). Different encoding strategies emphasize different biological properties: some capture mismatch patterns at specific nucleotide positions (e.g., NPM and Bulges encodings), whereas others focus on nucleotide identity and composition (e.g., One-Hot or K-mer encodings) (Hsu et al., 2013; Fu et al., 2013; Kim et al., 2015). These representations dramatically influence both prediction performance and interpretability.

Genome-editing applications have expanded significantly, motivating the need for predictive models that operate across editing modalities. Foundational studies like Yaish et al. (2022) focused on data processing and problem formulation for double-strand break knockout (KO); our study also incorporates Base Editing (BE) efficiency prediction on single-sequence data. By fusing engineered deaminases to Cas9 variants, CRISPR base editors introduce precise nucleotide substitutions without cleaving both DNA strands. Their efficiency depends on both gRNA sequence characteristics and the biochemical behavior of the deaminase component (Komor et al., 2016; Arbab et al., 2020; Koblan et al., 2018).

We used CHANGE-seq and GUIDE-seq datasets for the KO problem, which provide paired gRNA–off-target sequences and mismatch patterns, while we used the Hanna screening dataset for the BE problem, which consists of single gRNA sequences associated with viability-based z-scores. These scores, directly provided by the original authors and computed by their pipeline for experimental sequencing data analysis, quantify the biological impact of the editing on cell proliferation rather than directly measuring biochemical editing efficiency. This shift introduces a different and more constrained predictive setting (Marquart et al., 2021; Chen et al., 2023).

Our goal is to systematically evaluate the behavior of a single learning algorithm—XGBoost—across distinct CRISPR prediction tasks and dataset structures. Specifically, we assess how XGBoost performs on KO off-target prediction using GUIDE-seq and CHANGE-seq data, where paired gRNA–off-target sequences and mismatch-based encodings are available, and compare this behavior with its performance on BE efficiency prediction using the Hanna dataset, which relies exclusively on single gRNA sequences and continuous z-scores. For KO datasets, we evaluate XGBoost using eight established encoding strategies that capture positional, mismatch, and compositional features of paired sequences. For the Hanna dataset, we focus on two single-sequence encodings—One-Hot and K-mer representations—that are compatible with base editing data. This unified analysis allows us to examine the robustness, consistency, and interpretability of XGBoost across different CRISPR tasks, while highlighting how performance and feature-importance patterns depend on dataset structure and encoding choice. Specifically, we aim to determine:

(i) How interpretability tools such as SHAP and ALE reveal similarities or divergences between mechanisms.

(ii) Whether engineered features (GC-content, RNA structure, thermodynamic scores) improve BE prediction.

This paper is organized as follows: The background Sec. 2 introduces CRISPR–Cas9 knockout and base editing domains which motivates the role of feature representation in CRISPR modeling. Sec. 3 describes the materials and methods, including the datasets details and preprocessing process, which are reported in Sections 3.1 and 3.2, respectively. The details about feature encodings are reported in Sec. 3.3, while XGBoost model implementations are deeply reported in Sec. 3.4. A brief experimental setup is reported in Sec. 3.5, while hyperparameter optimization details are presented in Sec. 3.6. The results and discussions across both KO and BE domains are explained in Sec. 4, while the final conclusions of our work are reported in Sec. 5.

## 2 Background

One of the most important tools in contemporary genome engineering is the CRISPR–Cas9 system, which makes it possible to effectively disrupt, correct, and activate genes in a variety of biological systems. In CRISPR knockout, Cas9 endonuclease is guided to a complementary genomic location by a custom guide RNA (gRNA), causing a double-strand break (DSB) that is later fixed by endogenous DNA repair mechanisms (Jinek et al., 2012; Cong et al., 2013). Off-target cleavage, in which Cas9 binds and cuts regions bearing partial complementarity to the gRNA, may result in undesirable changes and potentially harmful effects, limiting CRISPR/Cas9 editing versatility (Kleinstiver et al., 2016).

Numerous biochemical and structural investigations have shown that Cas9 can withstand mismatches at particular locations along the gRNA–DNA hybrid, with the PAM-proximal “seed region” being essential for target recognition (Pacesa et al., 2022; Sternberg et al., 2015). High-throughput assays like GUIDE-seq, SITE-seq, and CIRCLE-seq, which map genome-wide cleavage profiles and give massive datasets for computational modeling, have been developed as a result of this positional sensitivity (Cameron et al., 2017; Tsai et al., 2015; 2017). These datasets have made it possible for the community to develop machine-learning algorithms that use mismatch patterns, sequence features, and other constructed representations to forecast off-target activity (Zhang et al., 2023).

Beyond DSB-dependent systems, a more recent method of genome engineering has been made possible by the development of CRISPR base editors (BEs), in which Cas9 variants coupled to cytidine or adenosine deaminases catalyze nucleotide conversions without generating DSBs (Gaudelli et al., 2017; Hua et al., 2022). Base editors add specific nucleotide changes without cutting both DNA strands, in contrast to CRISPR knockout systems that depend on double-strand DNA breaks. These approaches enable precise base conversions within a designated editing window by combining a deaminase enzyme with a catalytically inhibited Cas9 protein. Therefore, nucleotide position in relation to the editing window and PAM region, as well as sequence context, have a significant impact on base editing efficiency.

Base editing efficiency depends on factors such as gRNA structure, local chromatin accessibility, and the sequence preferences of the deaminase domain (Kim et al., 2024; Pallaseni et al., 2022).

Modeling CRISPR activity encourages the use of machine-learning techniques. Cas9 binding affinity, gRNA effectiveness, and off-target probability have all been predicted using traditional models like random forests and gradient boosting (Lin and Wong, 2018). Recently, a foundational study by Yaish et al. (2022) established a rigorous data processing and problem formulation pipeline for large-scale datasets like CHANGE-seq and GUIDE-seq, demonstrating that proper data transformation (e.g., logarithmic scaling) and the inclusion of inactive sites significantly boost XGBoost performance (achieving AUPRs up to ≈0.64 and Pearson correlations up to ≈0.76 ). Our work adopts this robust evaluation framework as a starting point to systematically investigate a different, yet equally critical aspect: how different feature encoding strategies impact both predictive accuracy and model explainability.

A critical aspect of this evaluation framework concerns the choice of performance metrics. Given the severe class imbalance characterizing both CHANGE-seq and GUIDE-seq (where inactive off-target sites outnumber active ones by orders of magnitude), the Area Under the Precision-Recall curve (AUPR) is the established primary metric for classification in this domain (Yaish et al., 2022), as it focuses exclusively on the minority class and is not inflated by the overwhelming number of true negatives that would otherwise bias accuracy or AUROC. Similarly, Pearson’s correlation coefficient is the standard measure for regression performance on cleavage counts, as it captures the linear agreement between predicted and observed activity levels on active sites. While additional metrics such as F1 score, precision, and recall are reported for completeness and to facilitate comparison with broader literature, their interpretation in highly imbalanced settings requires caution: a high recall at the cost of low precision, or *vice versa*, may reflect threshold sensitivity rather than true predictive power. Throughout this work, AUPR and Pearson correlation therefore serve as the primary indicators of model quality, consistent with the most directly comparable studies on these datasets.

Furthermore, while state-of-the-art deep learning approaches—such as GRU-based architectures (Yaish and Orenstein, 2024) or foundational models like DNABERT—have recently pushed predictive boundaries, gaining marginal AUPR improvements (typically 0.05−0.10 ) over tree-based ensembles, they often present severe interpretability issues (Konstantakos et al., 2022; Lazar et al., 2024). In contrast, XGBoost remains highly competitive while allowing for transparent, position-specific feature attribution. Indeed, a key finding from the recent literature is that feature representation is crucial: while more structured representations such as mismatch matrices, k-mer embeddings, or biophysical features can highlight regulatory patterns and positional dependencies, one-hot encodings capture local nucleotide identity (Verma, 2025; Wang et al., 2020).

Recent studies have shown strong performance of XGBoost-based approaches in CRISPR prediction tasks, including hybrid models such as CNN-XG for sgRNA on-target prediction Li et al. (2022), feature selection for deciphering Anti-CRISPR mechanisms (Ma et al., 2025) or anti-CRISPR protein identification (Zhu et al., 2022), and epigenetic analysis of features for CRISPR off-target activity (Mak et al., 2022). However, our study differs in focus. Instead of developing more complex architectures, we systematically evaluate different sequence encoding strategies within a unified XGBoost framework. In fact, choosing the most appropriate feature representation significantly affects the quality of the resulting models, whatever the method used (Kose et al., 2024). In addition, we address both knockout (KO) off-target prediction and base editing (BE) efficiency, whereas many previous works focus on a single task. We also incorporate SHAP analysis to improve model interpretability. Therefore, the novelty of this work lies in understanding how feature representation influences model performance across different CRISPR applications.

## 3 Materials and methods

### 3.1 Datasets

Our experimental framework utilizes weighted XGBoost models for both off-target event prediction and efficiency estimation. We employed three high-throughput datasets covering two distinct biological domains.

#### 3.1.1 Change-seq and GUIDE-seq (knockout domain)

Two datasets were used to examine off-target effects in the CRISPR/Cas9 system:

- **CHANGE-seq:** Includes 202,044 off-target sites across 110 (gRNAs) (Cameron et al., 2017).
- **GUIDE-seq:** Includes 1,072 off-target sites across 58 gRNAs (Tsai et al., 2015).

To train and test the models, we defined Active and Inactive Off-Target Sites (OTS) as follows:

- **CHANGE-seq:** Active OTS were defined as those with cleavage counts over 100, while sites with 100 or fewer counts were considered inactive. This resulted in **67,476 active** and **2,806,152 inactive** off-targets.
- **GUIDE-seq:** All experimentally identified off-targets were marked as active. Inactive OTS were identified using Cas-OFFinder as potential Cas9 mismatches (up to six mismatches), excluding the known active sites. This dataset comprises **1,702 active** and **1,476,301 inactive** off-targets.

#### 3.1.2 Hanna Dataset (base editing domain)

To extend the analysis to BE and to CRISPR screening task, we used the dataset from Hanna et al. (2021). This dataset contains 11,088 gRNAs targeting human single-nucleotide variations. Unlike the KO datasets, Hanna provides a single gRNA sequence associated with a z-score representing editing effects on cell proliferation.

### 3.2 Data pre-processing

Given the high variance in sequencing data and the severe class imbalance in the off-target datasets, specific pre-processing steps were applied to stabilize the model training.

**Logarithmic Transformation:** Specifically for the KO datasets (CHANGE-seq and GUIDE-seq), where the target variable represents raw cleavage counts, we applied a logarithmic transformation to reduce the impact of outliers and normalize the distribution. The transformation is defined as:

$$y^{\prime }=\log\left(y+1\right)$$

where y is the raw read count. Note that this transformation was not applied to the Hanna dataset, as its target variable (z-score) is already normalized.

**Class Weighting:** Both CHANGE-seq and GUIDE-seq datasets are highly imbalanced (approx. 97% negative). To prevent the model from biasing towards the majority class, we applied sample weighting during training. The weight for the active class was calculated as:

$$W_{\mathrm{active}}=\frac{\left|N_{\mathrm{inactive}}\right|}{\left|N_{\mathrm{inactive}}|+|N_{\mathrm{active}}\right|}$$

This ensures that both active and inactive samples contribute proportionally to the learning process.

### 3.3 Feature encoding strategies

Feature representation is central to our analysis. We categorize the encodings into two groups based on whether they explicitly model the alignment relationship between paired sequences or represent the nucleotide content directly.

#### 3.3.1 Alignment-based encodings (CHANGE-seq & GUIDE-seq)

These encodings are exclusively applicable to the paired datasets (KO task), as they rely on the explicit alignment between the gRNA and the Off-Target sequence to extract mismatch patterns. We evaluated:

- **Nucleotides to Position Mapping (NPM):** A 23×4×4 tensor representing the specific pairing between the gRNA nucleotide and the DNA off-target nucleotide at each position. This results in **368 features** that capture exactly which nucleotide substitution occurred (e.g., A-to-G vs. A-to-C).
- **Bulges Encoding:** An extension of NPM that accounts for DNA/RNA bulges (insertions/deletions) by adding a specific channel to the matrix, allowing the model to handle gapped alignments.
- **Label Encoding Pairwise (LEP):** Assigns a unique integer (0−15) to each of the 16 possible nucleotide pairs (e.g., A-A, A-T, G-C) at each position. This results in a compact vector of **23 features.**
- **Match-Mismatch (MM):** A binary vector of length 23 indicating simply the presence (1) or absence (0) of a mismatch at each position, ignoring nucleotide identity.
- **8XL Encoding:** A flattened concatenation of the One-Hot encoded gRNA and the One-Hot encoded Off-Target sequence, resulting in a vector of 23×8=184 features (Charlier et al., 2021).

#### 3.3.2 Sequence-content encodings

Unlike the alignment-based methods, these strategies focus on representing the nucleotide identity or compositional motifs directly. These encodings are versatile, as they were applied to the single-sequence Hanna dataset (BE) and adapted for the paired sequences in CHANGE-seq and GUIDE-seq (KO) through aggregation operations.

- **One-Hot Encoding (OH):** This strategy captures precise positional information. Each nucleotide is represented by a binary vector of length 4.
  • *For BE screening:* The gRNA is directly encoded, resulting in a flattened vector of 23×4=92features .
  • *For KO:* Both the target gRNA and the off-target sequence are encoded separately. The two resulting vectors are then combined via a **bitwise logical OR** operation. This allows the model to capture the presence of different nucleotides at the same position (effectively representing mismatches implicitly) while maintaining the same 92-feature dimensionality.
- **K-mer Encoding (KMER):** This strategy captures local sequence composition by counting the frequency of overlapping subsequences of length k=3 . This yields a vector of **63 features**, losing exact positional context but retaining motif information.
  • *For BE screening:* K-mers are counted on the gRNA sequence to capture motifs favored by the deaminase.
  • *For KO:* K-mers are extracted from the off-target sequence to represent the compositional context of the cleavage site.

In summary, how sequence information is maintained distinguishes these two representations from one another. By preserving the precise nucleotide identity at every site, one-hot encoding allows the model to discover biological patterns unique to each position. On the other hand, K-mer encoding captures motif composition but loses explicit positional information by summarizing sequences using the frequency of small subsequences.

### 3.4 Gradient boosting implementation

We implemented the models using XGBoost (eXtreme Gradient Boosting). This algorithm optimizes a loss function $L^{\left(t\right)}$ at iteration t using the second-order Taylor expansion:

$$L^{\left(t\right)}\approx \overset{n}{\underset{i=1}{\sum }}\left[g_{i}f_{t}\left(x_{i}\right)+\frac{1}{2}h_{i}f_{t}{\left(x_{i}\right)}^{2}\right]+\Omega \left(f_{t}\right)$$

where n is the total number of training samples, $x_{i}$ is the input feature vector for the i -th sample, and $f_{t}\left(x_{i}\right)$ represents the prediction of the new regression tree added at iteration t . The terms $g_{i}$ and $h_{i}$ are the first- and second-order derivatives (gradient and Hessian) of the loss function, respectively, and $\Omega \left(f_{t}\right)$ represents the regularization term (L1/L2) to control tree complexity and prevent overfitting. The overall ensemble model is updated iteratively:

$$F_{m}\left(x\right)=F_{m-1}\left(x\right)+\eta \cdot h_{m}\left(x\right)$$

where $F_{m}\left(x\right)$ is the cumulative prediction of the ensemble model at iteration m , $F_{m-1}\left(x\right)$ is the prediction from the previous iteration, $h_{m}\left(x\right)$ is the prediction of the individual weak learner (tree) added at the current step m (conceptually equivalent to $f_{t}$ in the optimization objective), and η is the learning rate that scales the contribution of each newly added tree.

### 3.5 Experimental setup and evaluation

#### 3.5.1 KO framework (CHANGE/GUIDE-seq)

We established two categories of predictive models for the KO datasets:

1. **Classification:** Aimed at determining whether an OTS is active or inactive.
2. **Regression:** Designed to estimate the exact cleavage count for an OTS.

For every encoding strategy, we trained two model variants to isolate the contribution of sequence information versus mismatch count:

- **Sequence-Only (-seq):** Uses only the encoded sequence features.
- **Sequence + Distance (-seq-dist):** Uses the encoded sequence features plus an explicit integer feature representing the Hamming distance (number of mismatches).

Cross-Validation Strategy:

- **CHANGE-seq:** We employed a “leave-11-gRNAs-out” approach. The 110 gRNAs were divided into 10 mutually exclusive sets. In each iteration, one set served as the test set.
- **GUIDE-seq:** We utilized a “leave-one-group-out” cross-validation, which iteratively leaves all the instances of a guide RNA as the validation set.

**Metrics:** For classification, we calculated the Area Under the Precision-Recall curve (AUPR). We specifically selected AUPR over the Area Under the Receiver Operating Characteristic (AUROC) curve due to the extreme class imbalance in our datasets (approximately 97% negative). Because AUROC incorporates True Negatives in its calculation, it can give an overly optimistic impression of performance on heavily skewed data. In contrast, AUPR, which focuses exclusively on Precision and Recall, provides a more rigorous and realistic evaluation of the model’s ability to identify rare active off-targets. For regression, we evaluated Pearson’s correlation coefficients on the read counts pertaining exclusively to active off-target sites.

#### 3.5.2 BE framework (hanna screening)

For the Hanna dataset, we maintained a similar dual-task approach but adapted for single sequences:

1. **Regression:** Predicting the continuous z-score directly (Metric: Pearson’s correlation).
2. **Classification:** Discretizing the z-score to distinguish highly effective gRNAs from the rest. We adopted the specific threshold of z<−2.0 to maintain methodological consistency with the original study (Hanna et al., 2021), where this cutoff was established.

Standard 10-fold cross-validation has been adopted for this dataset.

### 3.6 Hyperparameter optimization

All models were implemented in Python using the XGBoost and Scikit-learn libraries. Hyperparameter optimization was performed using RandomizedSearchCV, which randomly samples combinations of parameters from predefined ranges and evaluates them using cross-validation. This approach allowed us to explore a wide range of model configurations—adjusting parameters such as learning rate (η) , maximum tree depth, and regularization terms (λ,α) —without the computationally prohibitive cost of a full grid search, providing an efficient alternative to exhaustive GridSearch.

## 4 Results and discussion

### 4.1 XGBoost robustness on off-target prediction (KO)

We first evaluated XGBoost on the classical off-target prediction task. The use of XGBoost in this study is motivated by previous works showing that gradient boosting and other tree-based ensemble models provide strong predictive performance for CRISPR activity and gRNA efficiency prediction, particularly when modeling complex sequence–activity relationships. Tables 1, 2 summarize the classification and regression performance on GUIDE-seq and CHANGE-seq datasets, respectively. The reported metrics refer to the most predictive model variants, which incorporate both the sequence encoding and the explicit mismatch count feature (-seq-dist). Consistent with previous literature, encoding strategies that explicitly model mismatches (Bulges, NPM) perform strongly, achieving AUPRs of 0.555 and 0.568 respectively on GUIDE-seq.

**TABLE 1:** Classification performance across CHANGE-seq and GUIDE-seq datasets. Results refer to models incorporating both sequence and mismatch count features (-seq-dist).

CHANGE-seq						
Encoding	AUPR	F1	Accuracy	Precision	Recall	AUC
8XL	0.478±0.162	0.347±0.138	0.992±0.009	0.482±0.257	0.452±0.277	0.962±0.055
BULGES	0.612±0.165	0.388±0.187	0.990±0.008	0.365±0.254	0.709±0.229	0.978±0.046
K-MER	0.345±0.148	0.110±0.115	0.922±0.026	0.073±0.106	0.793±0.174	0.948±0.055
LEP	0.572±0.160	0.389±0.171	0.991±0.010	0.476±0.289	0.557±0.271	0.974±0.048
MM	0.345±0.148	0.110±0.115	0.922±0.026	0.073±0.106	0.793±0.174	0.948±0.055
NPM	0.611±0.164	0.387±0.186	0.990±0.008	0.365±0.255	0.708±0.229	0.978±0.046
OH	0.515±0.165	0.316±0.176	0.990±0.011	0.456±0.312	0.507±0.306	0.970±0.048
OH5C	0.564±0.167	0.354±0.169	0.992±0.009	0.494±0.304	0.519±0.291	0.973±0.048

GUIDE-seq						
Encoding	AUPR	F1	Accuracy	Precision	Recall	AUC
8XL	0.587±0.244	0.209±0.152	0.995±0.004	0.149±0.140	0.816±0.218	0.982±0.039
BULGES	0.555±0.190	0.113±0.111	0.988±0.009	0.066±0.073	0.906±0.133	0.984±0.039
K-MER	0.473±0.292	0.018±0.022	0.918±0.029	0.009±0.012	0.934±0.104	0.976±0.043
LEP	0.590±0.216	0.171±0.179	0.991±0.011	0.125±0.173	0.873±0.180	0.983±0.036
MM	0.473±0.292	0.018±0.022	0.918±0.029	0.009±0.012	0.934±0.104	0.976±0.043
NPM	0.568±0.198	0.117±0.118	0.988±0.009	0.069±0.078	0.907±0.128	0.984±0.038
OH	0.661±0.239	0.175±0.180	0.991±0.011	0.135±0.189	0.848±0.204	0.984±0.032
OH5C	0.608±0.230	0.189±0.166	0.993±0.007	0.136±0.154	0.834±0.199	0.983±0.037

**TABLE 2:** Regression performance across CHANGE-seq and GUIDE-seq datasets. Results refer to models incorporating both sequence and mismatch count features (-seq-dist).

CHANGE-seq			
Encoding	Pearson	Spearman	RMSE
8XL	0.502±0.258	0.081±0.080	103.747±124.521
BULGES	0.602±0.197	0.115±0.098	99.632±121.446
K-MER	0.315±0.116	0.099±0.068	133.110±114.326
LEP	0.484±0.265	0.091±0.090	101.595±123.507
MM	0.315±0.116	0.099±0.068	133.110±114.326
NPM	0.597±0.189	0.115±0.098	99.339±120.851
OH	0.526±0.253	0.088±0.083	103.421±124.108
OH5C	0.546±0.250	0.094±0.087	103.452±124.028

GUIDE-seq			
Encoding	Pearson	Spearman	RMSE
8XL	0.733±0.220	0.035±0.024	136.689±148.316
BULGES	0.692±0.214	0.039±0.029	129.582±144.914
K-MER	0.261±0.127	0.037±0.027	173.936±145.777
LEP	0.714±0.236	0.036±0.028	135.967±148.469
MM	0.261±0.127	0.037±0.027	173.936±145.777
NPM	0.678±0.228	0.039±0.029	128.186±144.276
OH	0.748±0.213	0.036±0.027	133.497±147.496
OH5C	0.756±0.197	0.036±0.028	131.150±146.134

However, a key observation is the performance of **One-Hot (OH)** encoding. On GUIDE-seq, OH achieves the highest AUPR of 0.661, notably outperforming K-mer encoding (0.473). This suggests that even without explicit mismatch features, XGBoost can learn the importance of specific nucleotide positions (e.g., the PAM-proximal region) directly from the raw sequence representation.

**FIGURE 1:**
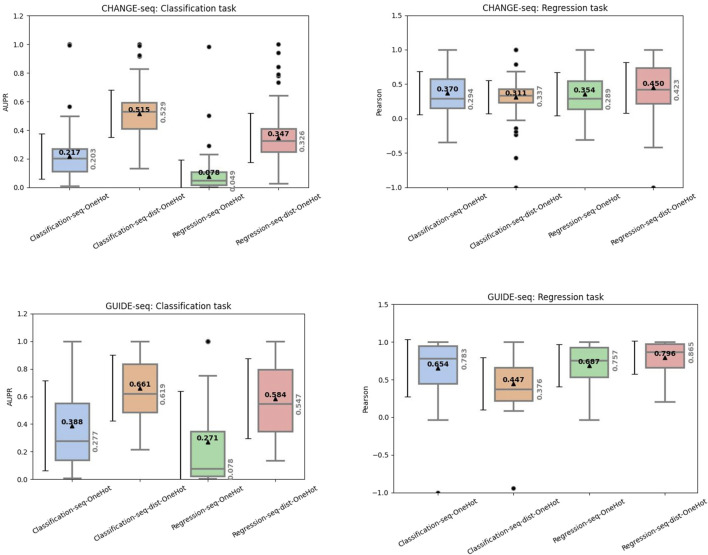
Performance distribution of XGBoost models trained with One-Hot encoding. The boxplots compare models using only sequence features (-seq) versus those including the mismatch count (-seq-dist).

To further investigate the model’s stability and the specific contribution of mismatch information, we analyzed the distribution of performance metrics across validation sets using One-Hot encoding (Figure 1); The boxplots reveal a crucial insight regarding the “Distance” feature. While sequence-only models (-seq) achieve respectable scores, the inclusion of the distance feature (-dist) yields a substantial performance boost, particularly in the classification tasks. For example, in the CHANGE-seq classification task (Figure 1A), the median AUPR increased from 0.217 to 0.515 by adding the distance feature. Similarly, for GUIDE-seq (Figure 1C), the median AUPR improved from 0.388 to 0.661. Beyond the median improvements, the inclusion of explicit mismatch counts consistently reduces the variance of the predictions (narrower interquartile ranges), suggesting that this feature acts as a robust regularizer, helping the model generalize better across diverse off-target samples where sequence context alone might be ambiguous.

### 4.2 XGBoost performance on base editing (hanna)

We then applied the XGBoost framework to the Base Editing task using the Hanna dataset. Given the single-sequence nature of this dataset, we focused our comparison on two sequence-content encodings: One-Hot vs. K-mer.

Hyperparameter optimization was applied to both encodings prior to evaluation.

#### 4.2.1 Hyperparameter tuning is critical for XGBoost

Optimization yielded substantial improvements for both encodings: One-Hot Pearson’s correlation increased from 0.671 to 0.746 (approximately 11% relative gain), while K-mer improved from 0.586 to 0.632.

#### 4.2.2 One-hot encoding consistently outperforms K-mer encoding across both predictive tasks

As summarized in Table 3, One-Hot models achieved substantially higher performance in both the classification task (AUPR of 0.723 vs. 0.517) and the regression task (Pearson’s correlation of 0.746 vs. 0.632) compared to K-mer models. This supports the hypothesis that Base Editing efficiency is highly position-dependent, a property better captured by the rigid spatial structure of One-Hot encoding than by the frequency-based K-mer approach, which aggregates motif counts irrespective of their location.

**TABLE 3:** Performance comparison of XGBoost models on the Hanna dataset (Base Editing) across classification and regression tasks.

Encoding	Classification (AUPR)	Regression (pearson)
One-hot	**0.723**	**0.746**
K-mer	0.517	0.632

Bold values indicate the best-performing results among the compared models.

These results confirm that for efficiency prediction tasks where no off-target context is available, the raw positional sequence information (One-Hot) combined with rigorous hyperparameter optimization provides the strongest predictive signal.

### 4.3 Implicit learning of biophysical properties

To further investigate the model’s capability to capture biological mechanisms, we tested whether explicitly providing biophysical features could enhance prediction accuracy on the Base Editing task (Hanna dataset). Specifically, following the methodology described in the DeepHF framework (Wang et al., 2019), we engineered a set of thermodynamic and compositional features, including:

- **GC Content:** The percentage of Guanine and Cytosine bases in the gRNA.
- **Melting Temperature** $\left(T_{m}\right)$ **:** The temperature at which the DNA double helix dissociates.
- **Gibbs Free Energy** (ΔG) **:** A measure of the thermodynamic stability of the RNA secondary structure calculated using RNAfold (Wang et al., 2019).

Our experiments demonstrated that adding these engineered features to the One-Hot representation yielded **no substantial improvement** in performance. On the contrary, we observed **marginal performance drops** when expanding the feature space, suggesting that these explicit descriptors likely introduced **noise or redundancy** rather than informative signal. This result is **noteworthy**: it indicates that the XGBoost model, when trained on raw sequence data (One-Hot), is sufficiently powerful to implicitly infer the necessary thermodynamic properties without the need for manual feature engineering.

### 4.4 Hyperparameter robustness and task consistency across base editors

To assess the robustness of our strategy, we evaluated whether the hyperparameters tuned on the primary dataset (BE39_MELJUSO) could be successfully transferred to other Base Editing contexts included in the Hanna dataset. It is important to clarify the experimental setup: while the model hyperparameters (e.g., learning rate, tree depth, regularization) were fixed to the values optimized for BE39_MELJUSO, we performed a full **retraining** of the XGBoost model for each specific target editor and cell line. This approach allows us to verify if the structural complexity learned for one Base Editor configuration is generalizable to others without requiring a new expensive search for hyperparameters.

#### 4.4.1 Performance stability

**FIGURE 2:**
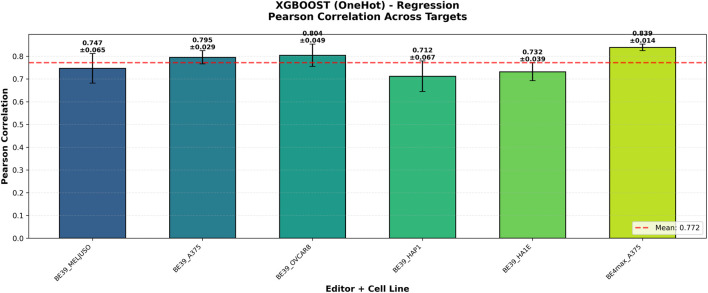
XGBoost (One-Hot) regression performance across targets. The bar chart shows the Pearson’s correlation of models trained and tested on various cell lines and editors. The model demonstrates strong generalization capabilities, maintaining high correlations across different Base Editor contexts (BE3.9, BE4max), confirming the robustness of the learned features.

As illustrated in Figure 2, the models demonstrated strong performance across all Base Editor targets. The regression models consistently maintained Pearson’s correlations >0.70 across different editors (BE39 and BE4max) and cell lines. This indicates that the optimal configuration found for the Meljuso cell line captures generalizable learning dynamics suitable for Base Editing data in general, and that One-Hot encoding provides a robust feature set for different deaminases.

#### 4.4.2 Consistency between tasks

**FIGURE 3:**
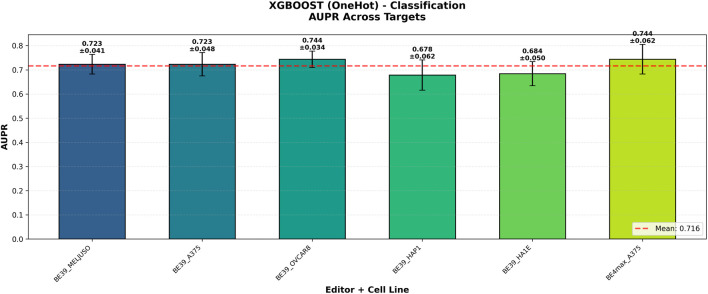
XGBoost (One-Hot) classification performance across targets. The classification model (AUPR) maintains strong performance across all targets. The results show strong alignment with the regression metrics, confirming that the model consistently identifies effective guides across different Base Editing contexts.

Furthermore, we analyzed the relationship between the Regression (predicting continuous z-score) and Classification (predicting active guides with z<−2.0 ) tasks. Our results show a high degree of consistency between the two formulations for Base Editing. As shown in Figure 3, targets that yielded high Pearson’s correlations in the regression task also achieved high AUPR scores in the classification setting. This alignment confirms that the predictive signal regarding editing efficiency is strong and stable, allowing XGBoost to effectively rank gRNAs regardless of whether the problem is framed as a continuous prediction or a binary selection. For both tasks, results have been evaluated according to standard metrics, and reported in Tables 4, 5.

**TABLE 4:** Regression performance across different targets.

Target	Pearson	Spearman	RMSE
BE39_MELJUSO	0.746±0.064	0.770±0.021	0.882±0.243
BE39_A375	0.795±0.029	0.772±0.019	0.738±0.108
BE39_OVCAR8	0.804±0.049	0.808±0.019	0.768±0.170
BE39_HAP1	0.712±0.067	0.751±0.026	1.022±0.271
BE39_HA1E	0.732±0.039	0.719±0.026	0.932±0.177
BE4max_A375	0.839±0.014	0.798±0.020	0.631±0.046

**TABLE 5:** Classification performance across different targets.

Target	AUPR	F1	Accuracy	Precision	Recall	AUC
BE39_MELJUSO	0.723±0.040	0.549±0.055	0.926±0.033	0.842	0.407	0.903±0.027
BE39_A375	0.723±0.048	0.549±0.044	0.936±0.021	0.840	0.408	0.938±0.016
BE39_OVCAR8	0.743±0.033	0.579±0.048	0.929±0.028	0.840	0.442	0.919±0.024
BE39_HAP1	0.678±0.062	0.531±0.040	0.915±0.036	0.834	0.390	0.881±0.031
BE39_HA1E	0.683±0.049	0.513±0.034	0.931±0.023	0.861	0.365	0.906±0.023
BE4max_A375	0.743±0.061	0.546±0.047	0.943±0.017	0.849	0.403	0.959±0.015

### 4.5 Error analysis: limits of regression at the extremes

**FIGURE 4:**
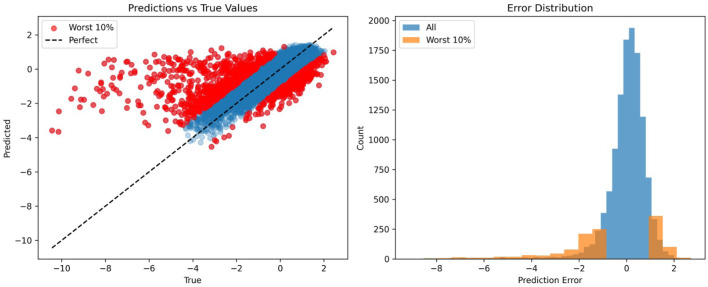
Analysis of Prediction Errors on the Hanna Dataset. The scatter plot (left) compares predicted vs. true z-scores, with the top 10% largest errors highlighted in red. The model struggles to predict extreme negative values (outliers <−4 ), showing a tendency to regress towards the mean due to data scarcity in the tails. The histogram (right) confirms that these outliers constitute the long tail of the error distribution.

To understand the performance ceiling of the regression model (Pearson ≈0.75), we analyzed the distribution of prediction errors. Figure 4 visualizes the relationship between predicted and true z-scores.

The scatter plot (Figure 4, left) reveals a distinct pattern: the model performs reliably within the standard range (z-scores between −2 and +2), where data is abundant. However, for the “Worst 10%” of predictions (highlighted in red), which correspond to extreme outliers (true z-scores <−4.0 ), the model consistently under-predicts the magnitude of the effect. Instead of predicting extreme negative values, the model regresses towards the mean.

This behavior is characteristic of gradient boosting algorithms when facing data scarcity in the tails of the distribution. Since high-efficiency guides are rare events, the model lacks sufficient training examples to learn the specific sequence rules governing these extreme phenotypes, preferring a conservative prediction. This limitation suggests that while the *ranking* of these guides remains generally correct (they are predicted as low, just not low enough), the *exact quantitative prediction* is inherently limited by the dataset distribution.

### 4.6 Explainability: impact of feature representation on model decisions

To verify that the models rely on biologically meaningful patterns rather than statistical artifacts, we employed a multi-faceted explainability strategy based on SHAP (SHapley Additive exPlanations) and Accumulated Local Effects (ALE).

#### 4.6.1 Local feature attribution (SHAP) in KO tasks

**FIGURE 5:**
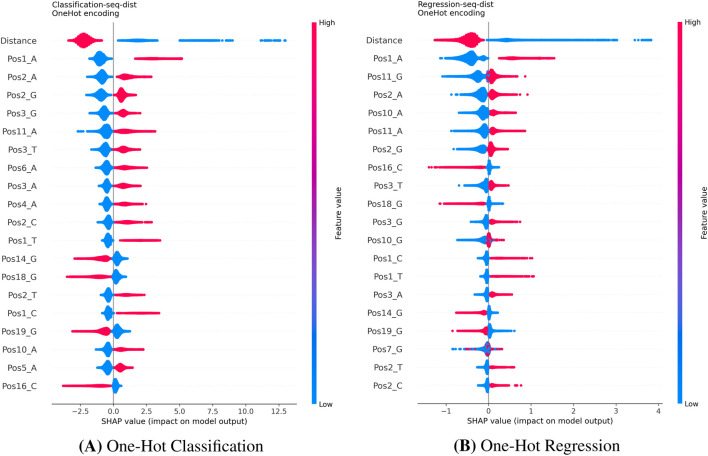
SHAP summary plots for One-Hot encoding on the CHANGE-seq dataset. Consistent with the biological mechanism, the ‘Distance’ feature dominates the model’s decision in both Classification **(A)** and Regression **(B)**. Beyond distance, the model relies on specific nucleotide positions (e.g., Pos1_A, Pos11_G) to fine-tune predictions. **(A)** Bulges Classification. **(B)** Bulges Regression.

**FIGURE 6:**
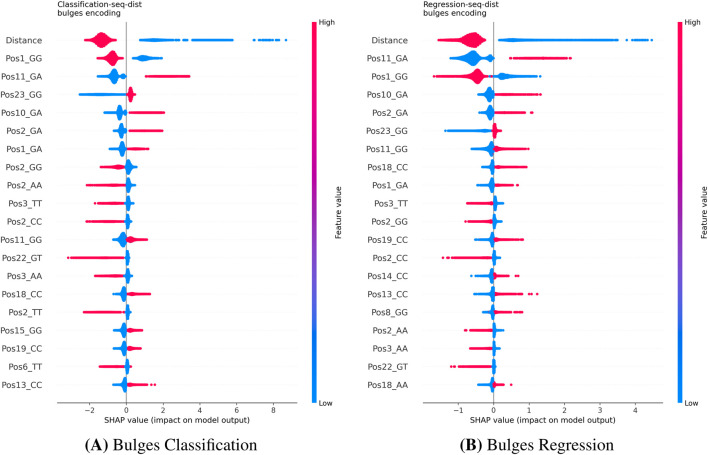
SHAP summary plots for Bulges encoding on the CHANGE-seq dataset. While ‘Distance’ remains the primary driver, the Bulges encoding enables the model to identify specific mismatch types (e.g., Pos1_GG, Pos11_GA) as critical features for both Classification **(A)** and Regression **(B)**, offering higher biological resolution than One-Hot. **(A)** K-mer Classification. **(B)** K-mer Regression.

**FIGURE 7:**
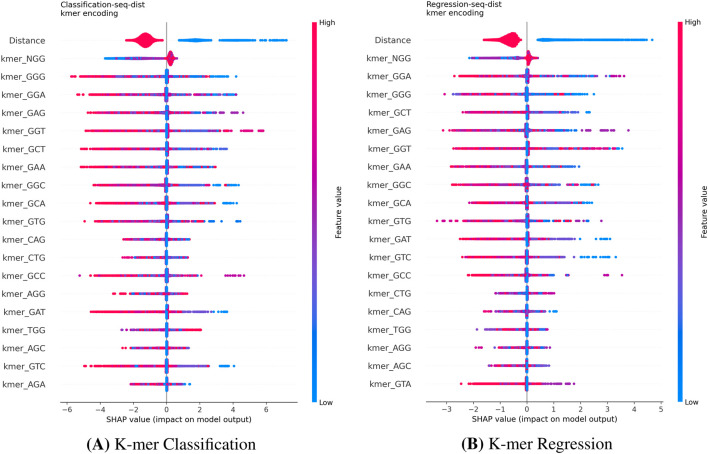
SHAP summary plots for K-mer encoding on the CHANGE-seq dataset. The K-mer models prioritize the global “Distance” feature, followed by compositional motifs (e.g., kmer_NGG, kmer_GGG), in both Classification **(A)** and Regression **(B)**. Unlike positional encodings, this representation emphasizes the frequency of subsequences in the off-target site.

For the CHANGE-seq and GUIDE-seq datasets, we used SHAP to derive local feature attributions. Although aggregating these local values into global importance metrics can be mathematically problematic (Bilodeau et al., 2024), our interpretation of the SHAP summary plots, which visualize the full distribution of local impacts, consistently identifies the **Distance** feature (mismatch count) as the dominant driver of model predictions across all encoding strategies (Figures 5–7). This analysis reveals that high distance values are associated with strongly negative SHAP values, indicating a pronounced reduction in predicted cleavage probability as the number of mismatches increases. This behavior is fully consistent with the established biological principle that increasing mismatches progressively destabilize Cas9–DNA binding.

#### 4.6.2 Quantifying the impact of mismatch count (ALE)

**FIGURE 8:**
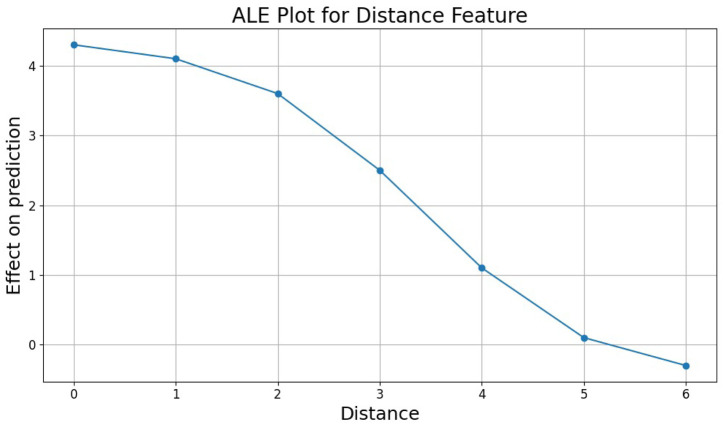
Accumulated Local Effects (ALE) analysis of the “Distance” feature. The plot quantifies the marginal effect of mismatch count on the prediction while accounting for feature correlations. It highlights the model’s sensitivity to even small numbers of mismatches (1–3), showing a sharp decline in predicted activity as genetic distance increases.

To isolate and quantify the effect of mismatches on KO prediction while accounting for feature correlations, we analyzed the *Distance* feature using ALE plots (Figure 8). The ALE plot reveals a clear trend: even small numbers of mismatches (distance 1–2) produce a measurable negative effect on the predicted score, which intensifies sharply as the distance increases. This confirms that the model has learned a stable and biologically plausible relationship between mismatch count and cleavage efficiency.

#### 4.6.3 Sequence-level interpretability and encoding effects (KO)

Beyond the global influence of the mismatch count, the choice of sequence encoding substantially affects the interpretability of learned patterns.

- The **One-Hot** models (Figure 5) highlight positional nucleotide effects, particularly at distal positions (e.g., position 1) and within the seed region, identifying specific bases that facilitate or hinder cleavage.
- The **Bulges** encoding (Figure 6) provides higher-resolution biological insight by explicitly modeling mismatch types. In both classification and regression tasks, the model identifies specific transitions (e.g., G → A at position 11) as highly influential features, capturing fine-grained thermodynamic effects accessible only through this representation.
- The **K-mer** encoding (Figure 7) shifts the focus from position to composition, prioritizing the frequency of specific subsequences (e.g., kmer_NGG) alongside the global distance feature.

#### 4.6.4 Interpretability on base editing (Hanna Dataset)

**FIGURE 9:**
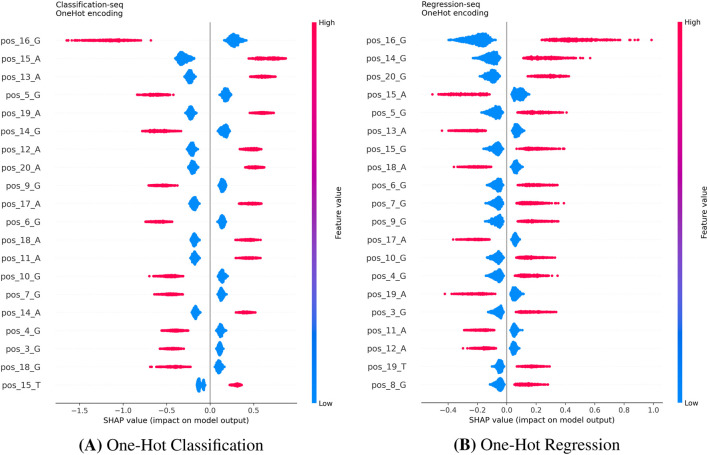
SHAP summary plots for One-Hot encoding on the Hanna dataset. Both the Classification **(A)** and Regression **(B)** models highlight specific nucleotide positions as key determinants (e.g., pos_16, pos_20). This confirms that the model captures the biological importance of the PAM-proximal seed region regardless of the task formulation. **(A)** K-mer Classification. **(B)** K-mer Regression.

**FIGURE 10:**
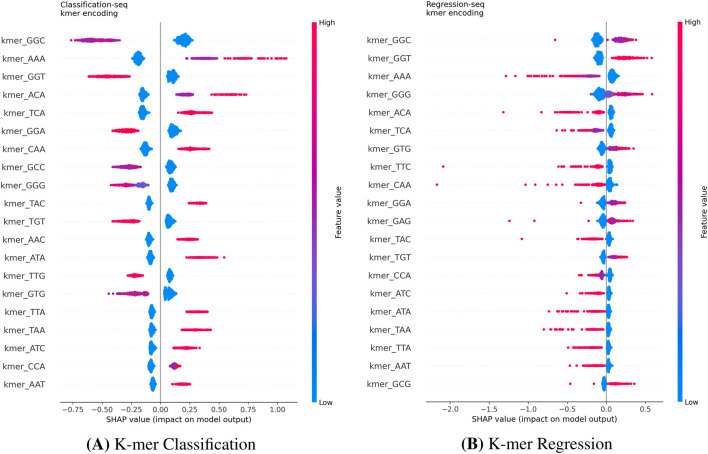
SHAP summary plots for K-mer encoding on the Hanna dataset. The K-mer models for Classification **(A)** and Regression **(B)** rely on the frequency of short subsequences (e.g., GGC, AAA). While capturing compositional motifs, this representation lacks the explicit positional resolution observed in the One-Hot models.

We extended the SHAP analysis to the Hanna dataset to compare how the model interprets single-sequence data for BE efficacy. Since the Distance feature is not applicable in this single-sequence context, the analysis focused purely on sequence content. The One-Hot models (Figure 9) clearly identify specific nucleotides in the PAM-proximal region (e.g., pos20A, pos17A) as key determinants across both classification and regression tasks. Conversely, the K-mer models (Figure 10) rely on the frequency of short subsequences (e.g., kmerGGC, kmerAAA). While K-mer encoding captures local composition, it loses precise positional information. This explains the superior performance of One-Hot encoding observed in Table 3: for base editing, the exact position of a nucleotide relative to the PAM is more predictive than its mere presence in a local motif.

## 5 Conclusion

In this study, we presented a comprehensive evaluation of XGBoost for CRISPR guide design, addressing both Off-Target prediction (KO) and Base Editing efficiency (BE). By testing on independent datasets (CHANGE-seq, GUIDE-seq, and Hanna) and optimizing for each domain, obtained three main results.

1. Encoding Strategy is Critical. More specifically, our results show that position-aware encodings such as Bulges and NPM achieve better performance for knockout (KO) off-target prediction, while One-Hot encoding performs better than K-mer encoding for base editing (BE) efficiency prediction on dataset Hanna.
2. XGBoost is Versatile. The algorithm performed robustly across different task formulations. Notably, we demonstrated a high degree of consistency between regression and classification tasks within the Base Editing domain. Targets that achieved high correlation in regression also showed high classification accuracy, confirming that the predictive signal is robust and that the framework can effectively rank guides regardless of the task formulation.
3. Explainability Validates Biology. Through SHAP analysis, we demonstrated that our models rely on biologically validated features, primarily the PAM-proximal seed region. This alignment between computational attribution and established molecular mechanisms strengthens the credibility of the framework and supports its application to guide RNA design tasks where interpretability is as important as predictive accuracy. Future work will focus on integrating these optimized representations into a unified tool for multi-modal CRISPR design.

The study also highlighted some limitations that could be addressed in future works. First, CRISPR off-target datasets are often highly imbalanced, which can affect prediction performance Yang et al. (2024). More specific techniques to ameliorate the quality of data can be considered to further improve the performance of the classification in terms of AUPR. Second, the current framework focuses mainly on sequence-based feature engineering and does not include other biological factors such as chromatin accessibility or epigenetic context that may affect editing outcomes. Further research would include biological factors with a systematic analysis of their impact on classification.

## Data Availability

Publicly available datasets were analyzed in this study. This data can be found here: https://github.com/ruth-hanna/base-editor-screen-analysis, https://github.com/OrensteinLab/SysEvalOffTarget.

## References

[B1] Anders C. Niewoehner O. Duerst A. Jinek M. (2014). Structural basis of pam-dependent target dna recognition by the cas9 endonuclease. Nature 513, 569–573. 10.1038/nature13579 25079318 PMC4176945

[B2] Arbab M. Shen M. W. Mok B. Wilson C. Matuszek Ż. Cassa C. A. (2020). Determinants of base editing outcomes from target library analysis and machine learning. Cell 182, 463–480. 10.1016/j.cell.2020.05.037 32533916 PMC7384975

[B3] Bilodeau B. Jaques N. Koh P. W. Kim B. (2024). Impossibility theorems for feature attribution. Proc. Natl. Acad. Sci. 121, e2304406120. 10.1073/pnas.2304406120 38181057 PMC10786278

[B4] Cameron P. Fuller C. K. Donohoue P. D. Jones B. N. Thompson M. S. Carter M. M. (2017). Mapping the genomic landscape of crispr–cas9 cleavage. Nat. Methods 14, 600–606. 10.1038/nmeth.4284 28459459

[B5] Charlier J. Nadon R. Makarenkov V. (2021). Accurate deep learning off-target prediction with novel sgrna-dna sequence encoding in crispr-cas9 gene editing. Bioinformatics 37, 2299–2307. 10.1093/bioinformatics/btab112 33599251

[B6] Chen T. Guestrin C. (2016). “Xgboost: a scalable tree boosting system,” in Proceedings of the 22nd acm sigkdd international conference on knowledge discovery and data mining, 785–794.

[B7] Chen X. Wang S. Chen Y. Xin H. Zhang S. Wu D. (2023). Non-invasive activation of intratumoural gene editing for improved adoptive t-cell therapy in solid tumours. Nat. Nanotechnol. 18, 933–944. 10.1038/s41565-023-01378-3 37188968

[B8] Cong L. Ran F. A. Cox D. Lin S. Barretto R. Habib N. (2013). Multiplex genome engineering using crispr/cas systems. Science 339, 819–823. 10.1126/science.1231143 23287718 PMC3795411

[B9] Fu Y. Foden J. A. Khayter C. Maeder M. L. Reyon D. Joung J. K. (2013). High-frequency off-target mutagenesis induced by crispr-cas nucleases in human cells. Nat. Biotechnology 31, 822–826. 10.1038/nbt.2623 23792628 PMC3773023

[B10] Gaudelli N. M. Komor A. C. Rees H. A. Packer M. S. Badran A. H. Bryson D. I. (2017). Programmable base editing of a• t to g• c in genomic dna without dna cleavage. Nature 551, 464–471. 10.1038/nature24644 29160308 PMC5726555

[B11] Hanna R. E. Hegde M. Fagre C. R. DeWeirdt P. C. Sangree A. K. Szegletes Z. (2021). Massively parallel assessment of human variants with base editor screens. Cell 184, 1064–1080. 10.1016/j.cell.2021.01.012 33606977

[B12] Hsu P. D. Scott D. A. Weinstein J. A. Ran F. A. Konermann S. Agarwala V. (2013). Dna targeting specificity of rna-guided cas9 nucleases. Nat. Biotechnology 31, 827–832. 10.1038/nbt.2647 23873081 PMC3969858

[B13] Hua K. Han P. Zhu J.-K. (2022). Improvement of base editors and prime editors advances precision genome engineering in plants. Plant Physiol. 188, 1795–1810. 10.1093/plphys/kiab591 34962995 PMC8968349

[B14] Jinek M. Chylinski K. Fonfara I. Hauer M. Doudna J. A. Charpentier E. (2012). A programmable dual-rna–guided dna endonuclease in adaptive bacterial immunity. Science 337, 816–821. 10.1126/science.1225829 22745249 PMC6286148

[B15] Kim D. Bae S. Park J. Kim E. Kim S. Yu H. R. (2015). Digenome-seq: genome-wide profiling of crispr-cas9 off-target effects in human cells. Nat. Methods 12, 237–243. 10.1038/nmeth.3284 25664545

[B16] Kim N. Choi S. Kim S. Song M. Seo J. H. Min S. (2024). Deep learning models to predict the editing efficiencies and outcomes of diverse base editors. Nat. Biotechnol. 42, 484–497. 10.1038/s41587-023-01792-x 37188916

[B17] Kleinstiver B. P. Pattanayak V. Prew M. S. Tsai S. Q. Nguyen N. T. Zheng Z. (2016). High-fidelity crispr–cas9 nucleases with no detectable genome-wide off-target effects. Nature 529, 490–495. 10.1038/nature16526 26735016 PMC4851738

[B18] Koblan L. W. Doman J. L. Wilson C. Levy J. M. Tay T. Newby G. A. (2018). Improving cytidine and adenine base editors by expression optimization and ancestral reconstruction. Nat. Biotechnology 36, 843–846. 10.1038/nbt.4172 29813047 PMC6126947

[B19] Komor A. C. Kim Y. B. Packer M. S. Zuris J. A. Liu D. R. (2016). Programmable editing of a target base in genomic dna without double-stranded dna cleavage. Nature 533, 420–424. 10.1038/nature17946 27096365 PMC4873371

[B20] Konstantakos V. Nentidis A. Krithara A. Paliouras G. (2022). Crispr–cas9 grna efficiency prediction: an overview of predictive tools and the role of deep learning. Nucleic Acids Res. 50, 3616–3637. 10.1093/nar/gkac192 35349718 PMC9023298

[B21] Kose A. M. Kocadagli O. Taştan C. Aktan C. Ünaldı O. M. Güzenge E. (2024). Unveiling off-target mutations in crispr guide rnas: implications for gene region specificity. CRISPR J. 7, 168–178. 10.1089/crispr.2024.0002 38922052

[B22] Lazar N. H. Celik S. Chen L. Fay M. M. Irish J. C. Jensen J. (2024). High-resolution genome-wide mapping of chromosome-arm-scale truncations induced by crispr–cas9 editing. Nat. Genetics 56, 1482–1493. 10.1038/s41588-024-01758-y 38811841 PMC11250378

[B23] Li B. Ai D. Liu X. (2022). Cnn-xg: a hybrid framework for sgrna on-target prediction. Biomolecules 12, 409. 10.3390/biom12030409 35327601 PMC8945678

[B24] Lin J. Wong K.-C. (2018). Off-target predictions in crispr-cas9 gene editing using deep learning. Bioinformatics 34, i656–i663. 10.1093/bioinformatics/bty554 30423072 PMC6129261

[B25] Ma Q. Zhang Y. Chen L. Bao Y. Guo W. Feng K. (2025). Machine learning-driven discovery of essential binding preference in anti-crispr proteins. PROTEOMICS – Clin. Appl. 19, e70013. 10.1002/prca.70013 40588792

[B26] Mak J. K. Störtz F. Minary P. (2022). Comprehensive computational analysis of epigenetic descriptors affecting crispr-cas9 off-target activity. BMC Genomics 23, 805. 10.1186/s12864-022-09012-7 36474180 PMC9724382

[B27] Marquart K. F. Allam A. Janjuha S. Sintsova A. Villiger L. Frey N. (2021). Predicting base editing outcomes with an attention-based deep learning algorithm trained on high-throughput target library screens. Nat. Communications 12, 5114. 10.1038/s41467-021-25375-z 34433819 PMC8387386

[B28] Pacesa M. Lin C.-H. Cléry A. Saha A. Arantes P. R. Bargsten K. (2022). Structural basis for cas9 off-target activity. Cell 185, 4067–4081. 10.1016/j.cell.2022.09.026 36306733 PMC10103147

[B29] Pallaseni A. Peets E. M. Koeppel J. Weller J. Vanderstichele T. Ho U. L. (2022). Predicting base editing outcomes using position-specific sequence determinants. Nucleic Acids Research 50, 3551–3564. 10.1093/nar/gkac161 35286377 PMC8989541

[B30] Saraswat P. Ranjan R. (2025). Unlocking the potential of crispr tools and databases for precision genome editing. Front. Plant Sci. 16, 1563711. 10.3389/fpls.2025.1563711 40761563 PMC12319022

[B31] Shrikumar A. Greenside P. Kundaje A. (2017). “Learning important features through propagating activation differences,” in International conference on machine learning (PMlR), 3145–3153.

[B32] Sternberg S. H. LaFrance B. Kaplan M. Doudna J. A. (2015). Conformational control of dna target cleavage by crispr–cas9. Nature 527, 110–113. 10.1038/nature15544 26524520 PMC4859810

[B33] Tsai S. Q. Zheng Z. Nguyen N. T. Liebers M. Topkar V. V. Thapar V. (2015). Guide-seq enables genome-wide profiling of off-target cleavage by crispr-cas nucleases. Nat. Biotechnology 33, 187–197. 10.1038/nbt.3117 25513782 PMC4320685

[B34] Tsai S. Q. Nguyen N. T. Malagon-Lopez J. Topkar V. V. Aryee M. J. Joung J. K. (2017). Circle-seq: a highly sensitive *in vitro* screen for genome-wide crispr–cas9 nuclease off-targets. Nat. Methods 14, 607–614. 10.1038/nmeth.4278 28459458 PMC5924695

[B35] Verma P. (2025). Hybrid neural networks for off-target prediction in gene editing: integrating sequence and biophysical features. Int. J. High Sch. Res. 7, 113–120. Available online at: https://terra-docs.s3.us-east-2.amazonaws.com/IJHSR/Articles/volume7-issue2/IJHSR_2025_72_113.pdf .

[B36] Wang D. Zhang C. Wang B. Li B. Wang Q. Liu D. (2019). Optimized crispr guide rna design for two high-fidelity cas9 variants by deep learning. Nat. Commun. 10, 4284. 10.1038/s41467-019-12281-8 31537810 PMC6753114

[B37] Wang J. Xiang X. Bolund L. Zhang X. Cheng L. Luo Y. (2020). Gnl-scorer: a generalized model for predicting crispr on-target activity by machine learning and featurization. J. Molecular Cell Biology 12, 909–911. 10.1093/jmcb/mjz116 31900489 PMC7883820

[B38] Yaish O. Orenstein Y. (2024). Generating, modeling and evaluating a large-scale set of crispr/cas9 off-target sites with bulges. Nucleic Acids Res. 52, 1612–1623. 10.1093/nar/gkae428 38813823 PMC11229338

[B39] Yaish O. Asif M. Orenstein Y. (2022). A systematic evaluation of data processing and problem formulation of crispr off-target site prediction. Briefings Bioinforma. 23, bbac157. 10.1093/bib/bbac157 35595297

[B40] Yang Y. Zheng Y. Zou Q. Li J. Feng H. (2024). Overcoming crispr-cas9 off-target prediction hurdles: a novel approach with esb rebalancing strategy and crispr-mca model. PLOS Comput. Biol. 20, e1012340. 10.1371/journal.pcbi.1012340 39226304 PMC11398643

[B41] Zhang S. Li X. Lin Q. Wong K.-C. (2019). Synergizing crispr/cas9 off-target predictions for ensemble insights and practical applications. Bioinformatics 35, 1108–1115. 10.1093/bioinformatics/bty748 30169558

[B42] Zhang G. Luo Y. Dai X. Dai Z. (2023). Benchmarking deep learning methods for predicting crispr/cas9 sgrna on-and off-target activities. Briefings Bioinforma. 24, bbad333. 10.1093/bib/bbad333 37775147

[B43] Zhu L. Wang X. Li F. Song J. (2022). Preacrs: a machine learning framework for identifying anti-crispr proteins. BMC Bioinforma. 23, 444. 10.1186/s12859-022-04986-3 36284264 PMC9597991

